# Effects of human disturbance on habitat and fish diversity in Neotropical streams

**DOI:** 10.1371/journal.pone.0274191

**Published:** 2022-09-09

**Authors:** Crislei Larentis, Bruna Caroline Kotz Kliemann, Mayara Pereira Neves, Rosilene Luciana Delariva

**Affiliations:** 1 Programa de Pós-Graduação em Biologia Comparada, Universidade Estadual de Maringá, Maringá, Paraná, Brazil; 2 Programa de Pós-graduação em Ciências Biológicas/Zoologia, Instituto de Biociências, Universidade Estadual Paulista (UNESP), Botucatu, São Paulo, Brazil; 3 Programa de Pós-graduação em Biologia Animal, Universidade Federal do Rio Grande do Sul, Porto Alegre, Rio Grande do Sul, Brazil; 4 Laboratório de Ictiologia, Ecologia e Biomonitoramentos (LIEB), Universidade Estadual do Oeste do Paraná – UNIOESTE, Cascavel, Paraná, Brazil; University of Eldoret, KENYA

## Abstract

Human pressures have been intensely modifying freshwater ecosystems worldwide. We assessed the effects of human pressure on habitat diversity and primary productivity to understand the consequences on fish fauna in 25 tropical and subtropical streams of two globally important ecoregions: Iguassu and Upper Paraná. We hypothesized that the increased human pressure (urbanization and agriculture) on stream environments, both at the local and catchment scales, directly decreases habitat diversity. We also hypothesized that increased human pressure triggers changes in primary productivity and fish fauna composition and structure. We evaluated the human pressure intensity using the Integrated Disturbance Index and the Rapid Habitat Diversity Assessment protocol, which combines information about land use, land cover and environmental characteristics of the stream catchment and sampling sites. Streams with increased human disturbance had lower habitat diversity, higher primary productivity, and high non-native species abundance. Fish compositional turnover was associated with increased human disturbance. Native and degradation-sensitive fish species, especially endemic ones, were associated with streams with higher habitat diversity and forested cover. Degradation-resistant fishes, mostly non-native species, were associated with streams with higher human disturbance and urban land use. Although human pressure did not affect species richness, Shannon diversity, and Simpson dominance, there were significant effects on numerical abundance and fish species equitability. In this study, human pressure directly affected habitat structure, with indirect consequences for fish fauna, increasing the potential for local extirpation of rare species.

## Introduction

The increase in the human population and the demand for products and services have caused numerous environmental disturbances that strongly affect freshwater ecosystems [1–3]. In rivers and streams, changes in land cover, boosted by agricultural development and urban expansion, are main drivers of environmental degradation [4, 5]. Habitat diversity, hydrology, water quality, productivity, and freshwater biodiversity are all threatened [6, 7]. Furthermore, human activities are responsible for the introduction of non-native fish species into diverse freshwater environments. This introduction can promote changes in the population dynamics of native species due to competition for food and habitat besides the proliferation of diseases [8, 9].

Changes in land cover in stream catchments cause alterations in both the riparian zone and instream habitats, which can lead to habitat homogenization [10], severely affecting the aquatic biota [11]. Erosion and sedimentation [12], soil compaction affecting water infiltration [13, 14], and streambed channeling [15] have been widely observed in stream ecosystems. These physical alterations lead to habitat homogenization, low diversity of food resources, and changes in the structure of the fish fauna [16–18]. Environmental heterogeneity and microhabitat diversity are fundamental to the availability of shelter and food resources for fish species [19]. These conditions facilitate the existence of diverse species in these streams through utilization of resources in different microhabitats [20, 21]. The increased input of nutrients in the water column resulting from urban and agricultural land use causes changes not only in water physic-chemical conditions but also in terms of primary productivity and aquatic biota [22–24]. Nutrient enrichment owing to effluent discharge can intensify biological activity and drastically alter the composition and structure of aquatic food webs. One of the main changes is increased chlorophyll-**α** (Chl-**α**) biomass [25], which is widely used to measure eutrophication [26].

Effluent discharge or leaching is much more intense in urban streams, where eutrophication is common [23], and can be a consequence of the precariousness of sewage disposal, as documented in Brazil [27, 28]. Illegal discharge of industrial and domestic sewage in watercourses [29], and rainwater runoff also contribute to this process [30]. Eutrophication not only affects freshwater biodiversity but also human health and ecosystem services [31].

Another worrying factor is the introduction of non-native fish species. This is also considered an important stressor for native assemblages in freshwater environments worldwide [32–34]. In disturbed water courses, non-native species introductions are mainly a result of activities related to aquaculture and aquarism [9, 35]. The establishment of non-native fish species can lead to changes in species composition [34]. These changes are related to an increase in the dominance of more degradation-resistant species, and a decrease and/or loss of species diversity [36]. Over time, these processes can induce fish fauna homogenization, with a global trend toward biotic homogenization [34].

Neotropical streams shelter the world’s highest richness and endemism of fishes [37] and these characteristics are especially relevant in two ecoregions in southern Brazil—Iguassu and Upper Paraná. Such conditions are a result of rapids and waterfalls that occur within these basins, which limit fish distribution upstream, contributing to the high level of endemism in these ecoregions [38]. Thus, evaluating fish species composition and structure of these ecoregions is important in understanding biogeographic aspects and factors that can affect species distribution. Despite their exceptional diversity and endemism, the streams and tributaries of the Iguassu and Upper Paraná ecoregions have undergone intense anthropogenic transformations. Thus, there is an urgent need to obtain information on fish fauna in headwater streams in the Iguassu and Upper Paraná ecoregions.

In this study, we aimed to assess the effects of human pressure on habitat diversity, primary productivity, and fish fauna composition and structure in 25 Neotropical streams in southern Brazil. We hypothesized that increased human pressure on stream environments, both locally and at catchment scales, decreases habitat diversity and triggers changes in primary productivity, fish species composition, and assemblage structure. We tested the following predictions: i) there is an inverse relationship between habitat diversity and human pressure according to the integrated disturbance index (IDI); ii) streams with low habitat diversity and intense disturbance have higher primary productivity; iii) degradation-resistant species, including non-native ones, are indicators of disturbed streams, and degradation-sensitive and endemic species are indicators of less disturbed streams; iv) species restrictedness highlights endemic and rare species occurring in streams closer to natural conditions; and v) numeric abundance, species richness, and dominance increase with disturbance intensification, and species diversity and equitability decrease in response to this intensification. Considering the regional pool of species, we expect native and endemic species to display specific requirements regarding food, habitat, and ecological conditions. Understanding how human pressure affects stream environments provides useful information for conservation efforts, particularly for endemic species.

## Material and methods

### Ethics statement

This study was carried out in strict accordance with protocols in their ethical and methodological aspects for the use of fish. The protocol was approved by the Committee on the Ethics of Animal Experiments of the Universidade Federal do Rio Grande do Sul (Protocol Number CEUA– 32,734). The fish sampling was conducted under license from the Instituto Chico Mendes de Conservação da Biodiversidade (ICMBio) (Number processes: 25039; 27252). Regarding access to sampling sites, permission was only requested from Instituto Chico Mendes de Conservação da Biodiversidade of the Paraná State for sampling in the Rebio das Perobas; for all the other sites, permission was granted by the private owners.

### Study area

The study area comprised the Iguassu and Upper Paraná ecoregions, which are globally important because of their species richness and endemism [38]. The Iguassu ecoregion includes the Iguaçu River Basin and all its tributaries in Brazil above the Iguaçu Falls [38]. The Upper Paraná ecoregion includes the drainage basin of the Upper Paraná River (comprise Piquiri and Ivaí Basins) and its tributaries above the former Guaíra Falls (Salto de Sete Quedas) [37].

**Fig 1:**
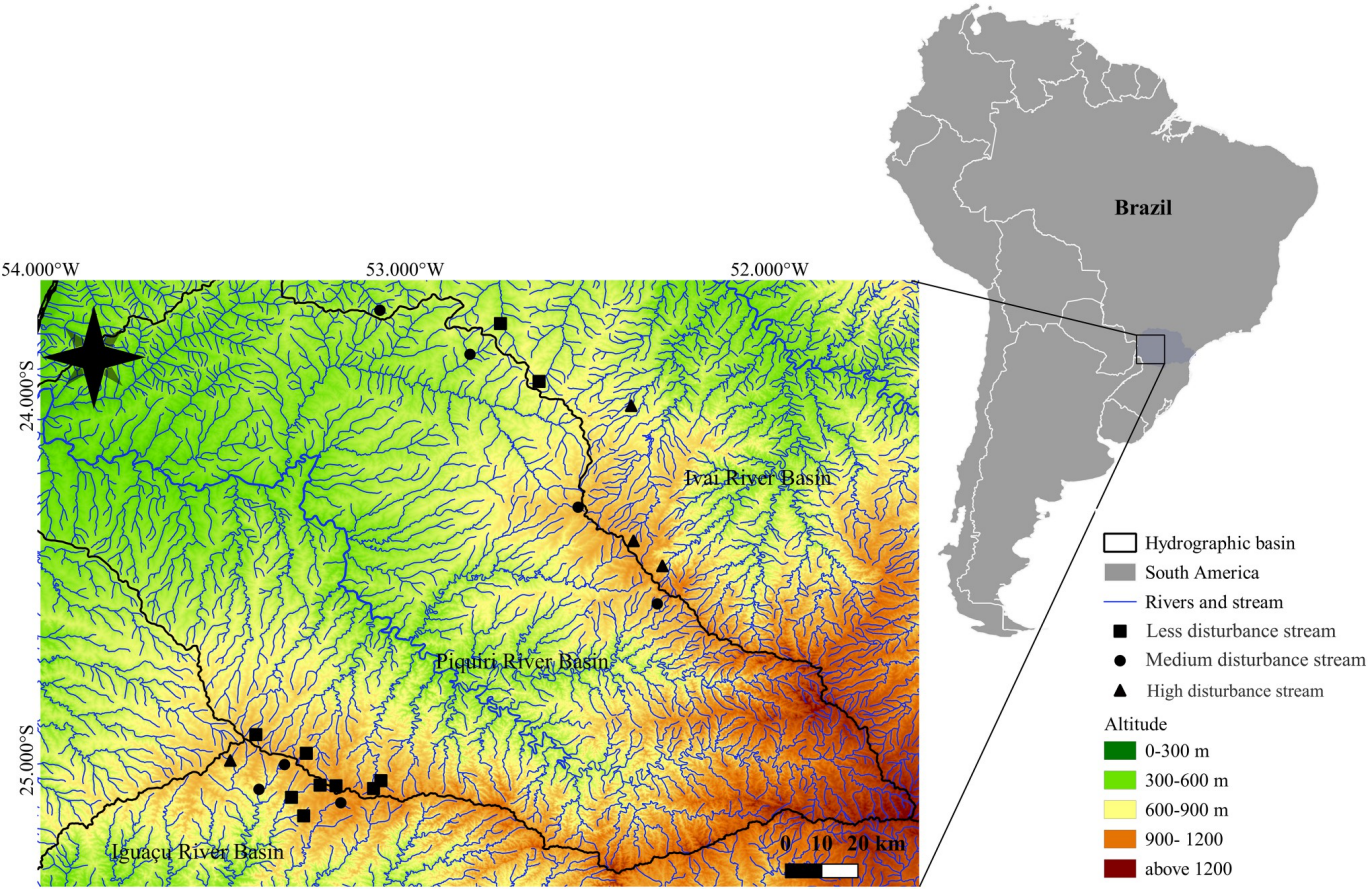
Study area: Sampling sites distributed in the Iguaçu, Piquiri, and Ivaí River Basins, Brazil. The classification of streams as high, medium and low disturbance was according to the Integrated disturbance index values: < 0.09 values—low disturbance (sites with conditions closer to the natural), 0.10 to 0.19—medium disturbance (altered sites), > 0.2—high disturbance (extremely impacted sites). “Raster data obtained from EMBRAPA (Intellectual Property Rights—US Geological Survey), accessed on March 20, 2022. https://www.cnpm.embrapa.br/projetos/relevobr/download/pr/pr.htm”.

The Iguaçu (54.820 km^2^), Piquiri (24.171,70 km^2^), and Ivaí (36.540 km²) river basins [39] (Fig 1) are in a region of a humid, subtropical climate (Cfa), as defined by the Köppen climate classification [40], with hot and humid summers and cold winters. The average annual precipitation varies between 1100 and 2000 mm and the average annual temperatures vary between 11.5 and 25°C [38]. The Iguaçu River originates in the Serra do Mar and travels across the Paraná Plateau before dropping off at Iguaçu Falls near its confluence with the Paraná River. The altitude varies between 908 m (origin) and 78 m (outfall in the Paraná River) above sea level, with numerous rapids and falls present along its course [41]. The Piquiri River originates in the Serra do São João at 1237 m altitude, on the third plateau in the south-central region of the state and runs 485 km before reaching the Paraná River [42]. The Ivaí River is a left-bank tributary of the Paraná River in Paraná State [42]. This river is formed in the municipality of Prudentópolis by the confluence of the Patos and São João rivers, both in the State Park of Serra da Esperança, on the border between the second and third plateaus of Paraná State [42]. In these three basins, the predominant land use is livestock pasture and agriculture, with the cultivation of cereals (soybean, corn, wheat) and sugarcane in the sandy soils. The industrial activities are also directly related to agriculture in the interior of Paraná State [39].

A total of 25 streams were sampled (Fig 1; S1 Table) in the Iguaçu (nine streams), Piquiri (ten streams), and Ivaí (six streams) river basins. Sampled streams ranged in size from 1st to 3^rd^ order [43] and in land cover gradient from 3 to 80% of reduction of native forest cover.

### Land use and land cover characterization

We calculated the different land use and land cover by demarcating the catchment above the sampling point for each stream. The geographical coordinates of the sampling sites were input into Quantum Geographic Information System (QGIS) software (QGIS version 2.18.10). A digital elevation model (DEM) was downloaded from the EMBRAPA Monitoramento por Satélite (https://www.cnpm.embrapa.br/projetos/relevobr/download/pr/pr.htm) [44]. Using the GRASS plugin in QGIS, the DEM raster was opened, and the catchment area for each sampling site was delimited with the ‘r.watershed’ and ‘r.water.outlet’ tools. The land use and land cover data (from 2017) were downloaded from the MapBiomas website (https://mapbiomas.org/colecoes-mapbiomas-1?cama_set_language=pt-BR). This raster was used as a base to calculate the different land use and land cover within the polygon of each catchment delimited for the sampling sites. The area (km²) of the following land uses was calculated: urbanized area—paved area, residential and industrial area; agricultural area which included pastures, plantations of annual and perennial crops and silviculture. In relation to land cover, the forested area included areas of riparian forest and remnants of native forest.

### Habitat diversity

We applied the rapid habitat diversity assessment (RHDA) protocol, adapted by Callisto *et al*. [45], to characterize the habitat diversity of the streams. For this, the Rapid Assessment Protocol (RAP’s) was used, which is a cost-effective bioassessment method because it allows integrated analysis of stream ecosystems through visual inspection of the area. This RAP captures the characteristics of the habitat for rating the degree of impact measured in set scores, determining environmental quality, and indicating the cumulative impacts of multiple stressors [45]. The RHDA protocol consists of 22 parameters (detailed in the S1 File). The first 10 were adapted from the Ohio Environmental Protection Agency—USA [46] and analyze the signals of human pressures in the reach characteristics. The other parameters were adapted from the protocol presented by Hannaford *et al*. [47] and assess the environmental characteristics of the sampled site. We used the total score obtained in the RHDA protocol to represent habitat diversity at the sampled sites.

### Integrated Disturbance Index

We calculated the Integrated Disturbance Index (IDI) to describe the intensity of human pressure in the sampled streams. For this purpose, we calculated two indices: one at the local scale (sampling sites), the Local Disturbance Index (LDI), and one at the catchment scale (stream catchment), the Catchment Disturbance Index (CDI). LDI was calculated using the *W1_ hall* metric according to Kaufmann *et al*. [48] and Ligeiro *et al*. [1]. Eleven types of disturbances were evaluated for the LDI, counted by observations in the stream and riparian zone: buildings, channel revetment, pavement, roads, pipes, trash and landfill, parks and lawns, row crop agriculture, pasture, logging, and mining. To measure these disturbances, each sampled reach of the stream was divided into five transects. The obtained values were weighted according to their proximity to the observation point inside the stream’s channel, where 0 represented absence of disturbance; B, inside the channel or in the margin; C, disturbance in less than 10 m; and P, disturbance in more than 10m [48]. We calculated the CDI considering the land use percentages calculated for each sampled streams’ catchment (section “Land use and land cover characterization”), according to Ligeiro *et al*. [1] (adapted from Rawer-Jost *et al*. [49]):

$$\text{Catchment}\;\text{Disturbance}\;\text{Index}\left(\text{CDI}\right)\text{=}4\;\text{x}\;\%\text{urban}\;\text{areas}+2\;\text{x}\;\%\;\text{agricultural}\;\text{areas}+\text{pasture}\;\text{areas}$$ (1)

The CDI values range from 0 (no land use in the catchment) to 400 (entire catchment occupied by urban and/or agricultural areas).

Finally, we summarized these two indices (LDI and CDI) in the IDI [1], applying the following formula:

$$\text{Integrated}\;\text{Disturbance}\;\text{Index}\left(\text{IDI}\right)={\left[{\text{(}\frac{\text{LDI}}{5}\text{)}}^{2}+{\text{(}\frac{\text{CDI}}{300})}^{2}\right]}^{1/2}$$ (2)

This index ranges from 0 to 1, and values close to 1 indicate major disturbances inside the stream channel, in the riparian zone, and/or in the catchment of the sampled sites. Like RHDA, in terms of the IDI values, the stream disturbances were classified into three IDI levels: < 0.09, low disturbance (sites with conditions closer to natural); 0.10–0.19, medium disturbance (altered sites); and > 0.2, high disturbance (extremely impacted sites). We considered high disturbance at IDI > 0.2 because this value included streams with urban land use greater than 20% in their catchment and human interference on the banks and stream channel.

### Primary productivity

We used the Chl-α biomass to evaluate primary productivity. Chl-α concentration is an accepted indicator of eutrophication that can be examined to assess if the input of anthropogenic nutrients is affecting an ecosystem [50]. Chlorophyll-α is a primary indicator and can respond to increasing inputs of nutrients before more serious and irreparable damage occurs, such as loss of submerged aquatic vegetation [51]. Herein, the Chl-α biomass (μg/L^-1^) was determined from water samples collected at each sampling site. After sampling, 1 L of water from each sample was filtered by a vacuum pump using a fiberglass filter (Merck^®^, GF-47 mm). The filters with the retained particles were analyzed in the laboratory using the parameters described for limnological analysis [52].

### Fish assemblage sampling

To verify the composition and diversity of the fish fauna, we sampled three occasions (March—April 2017; July—August 2017, and December 2017—January 2018). In each stream, we conducted fish sampling 50 m reaches using three-pass electrofishing with 40 minutes of effort for each pass. To prevent fish escape, we delimited the reach using blocking nets. After capture, the fish were anesthetized and fixed in 10% formaldehyde. In the laboratory, individuals were identified according to specific identification keys [41, 53, 54]. This study was carried out in strict accordance with protocols in their ethical and methodological aspects for the use of fish. We deposited specimens of all the sampled species in the Coleção Ictiológica do Nupélia (Núcleo de Pesquisas em Limnologia, Ictiologia e Aquicultura, Universidade Estadual de Maringá—UEM, Paraná State), and in the Coleção Ictiológica of the Universidade Federal do Rio Grande do Sul (UFRGS, Rio Grande do Sul State). The species list with respective vouchers is available only in the online version (S2 Table). We also classified the species as native and non-native in each sampling basin (Iguaçu, Piquiri, and Ivaí) according to Baumgartner *et al*. [41], Graça, Pavanelli [53], and Ota *et al*. [54] (S2 Table).

### Statistical analysis

First, we assessed the effects of human pressure (agriculture and urbanization), represented here by the disturbance indices, on environmental conditions of the streams, as portrayed by the physical characteristics of the sampling sites. To investigate the correlation among habitat diversity, IDI, land use, land cover and their possible effects on local primary productivity, we applied Spearman’s correlation analysis using *corrplot* [55] and *Hmisc* [56] packages. Considering the different scales of variables, the variables were log-transformed with the ‘log’ function. These preliminary analyses are fundamental to understanding how environmental variables interact and avoiding collinearity in the subsequent analysis. Then, how changes in the environmental characteristics of streams affected the fish faunal composition and species distribution and what species would be good indicators of the different stream groups were determined.

To test the influence of environmental variables (explanatory variables) on the spatial distribution of species (response variables), we used distance-based redundancy analysis (dbRDA) [57], based on the Bray-Curtis distance. This analysis is a form of multivariate multiple regression used to assess the relative importance of each explanatory variable in explaining the differences between the response variables. For this purpose, a square root transformation on the species abundance data was used, thus reducing the weight of the most abundant species in the analysis. The environmental variables were log-transformed at the different scales. To ensure the effectiveness of the variables in the analysis, environmental variables were selected using two criteria. First, all highly correlated variables (Spearman’s r ≥ 0.7, p < 0.05) [58] were excluded. Second, the variance inflation factor (VIF) was applied to the variables selected by Spearman’s correlation and those with VIF > 10 were excluded [59]. For VIF > 10, there was severe multicollinearity requiring correction. Nitrate, phosphate, and total nitrogen were excluded due to this process. After the selection of environmental variables, we performed dbRDA with the ‘capscale’ function in the *vegan* package [60]. The statistical significance of dbRDA was assessed using a permutation test for dbRDA, using the ‘anova.cca’ function, with 999 permutations, of the *vegan* package [60].

To determine whether there were fish species that could be an indicator for each site category according to the IDI levels (> 0.09—low disturbance, 0.10 to 0.19 –medium disturbance, and < 0.2—high disturbance), indicator value analysis was applied (IndVal) [61]. Indicator values reflect specificity, i.e., the probability of a taxon occurring in a group, and fidelity, i.e., the relative abundance of the taxon in that group. The method of Dufrêne, Legendre [61] calculates the IndVal index between the species and each site group and then looks for the group corresponding to the highest association value. Finally, the statistical significance of this relationship is tested using a permutation test. IndVal is the default index used to measure the association between a species and a group of sites in ‘multipatt’. However, by default ‘multipatt’ uses an extension of the original Indicator Value method, because the function looks for indicator species of both individual site groups and combinations of site groups, as explained in De Cáceres *et al*. [62]. IndVal produces an indicator species value (ISV) that ranges from 0 (absent) to 1 (present in all samples of a particular group). Species that are considered the “best” indicators of a group are those with scores closest to 1, indicating that they are found within their group only and do not occur anywhere else. IndVal is based on the numerical abundance of fish species and was calculated using the ‘multipatt’ function, with 999 permutations, in the *indicspecies* package version 1.7.8 [62].

Restrictedness was also calculated using the ‘restrictedness’ function in the *funrar* package [63]. This taxonomic metric indicates the presence of rare species at the regional level. The calculation produces a single index per species and is based on a complete dataset containing the presence-absence or relative abundance of species at each site [63]. Here, we calculated the restrictedness metric using the relative abundance of the species. We measured the numerical abundance (number of individuals by species) and species richness (species number by stream) to calculate the taxonomic diversity indices (Simpson dominance, Joule equitability, and Shannon diversity). We calculated the taxonomic diversity indices usingthe *BiodiversityR* and *vegan* packages [60, 64]. These indices are based on numeric abundance and facilitate the detection of changes in fish assemblages related to alterations in species abundance and are a useful tool to investigate the effects of human pressure on fish assemblage structure [65]. The next step was to perform Generalized Linear Mixed Models (GLMMs) with Simpson dominance, Joule equitability, Shannon diversity, the numerical abundance of species, and species richness as response variables, with IDI and the proportion of the numeric abundance of the non-native per native fish species (NNAbu, non-native species abundance/native species abundance) as fixed factors, and basin as a random factor. The NNAbu was included because the presence of non-native species is one of the consequences of human pressure on freshwater environments and has caused numerous alterations in native assemblages [9]. According to previous correlation analysis results, habitat diversity and IDI are significantly correlated, indicating that only one of these variables should be used in the models. The IDI was used because this index represents the human pressure in the local scale (riparian area) and regional scale (catchment) of the streams. The explanatory variables were log-transformed to standardize the scales. We checked the proper family distribution for each response variable using the function ‘fitdist’ from fitdistrplus package [66]. Subsequently, GLMMs with beta family distributions were run for Simpson’s dominance and Joule equitability (values bounded between 0 and 1) using the ‘glmmTMB’ function from glmmTMB package [67]. For Shannon diversity, species richness, and numerical abundance, GLMMs with Gaussian family distribution were run using the ‘lmer’ function from the lme4 package [68]. Models with an interaction between the effect factors (IDI and NNAbu) and models without interaction were compared using ANOVA to determine if there were differences between the tested models. Additionally, the Akaike information criterion (AIC) [69] was used to select the best model among the tested models for each response variable [70]. The residual plots were visually inspected to check the model assumptions and the plots of the models were built using the ggplot2 package [71].

All analyzes were performed in R programming environment (ver. 3.2.3, R Foundation for Statistical Computing, Vienna, Austria). The level of statistical significance for all analyses was p< 0.05.

## Results

### Effects of the human pressure on environmental characteristics of streams

**Fig 2:**
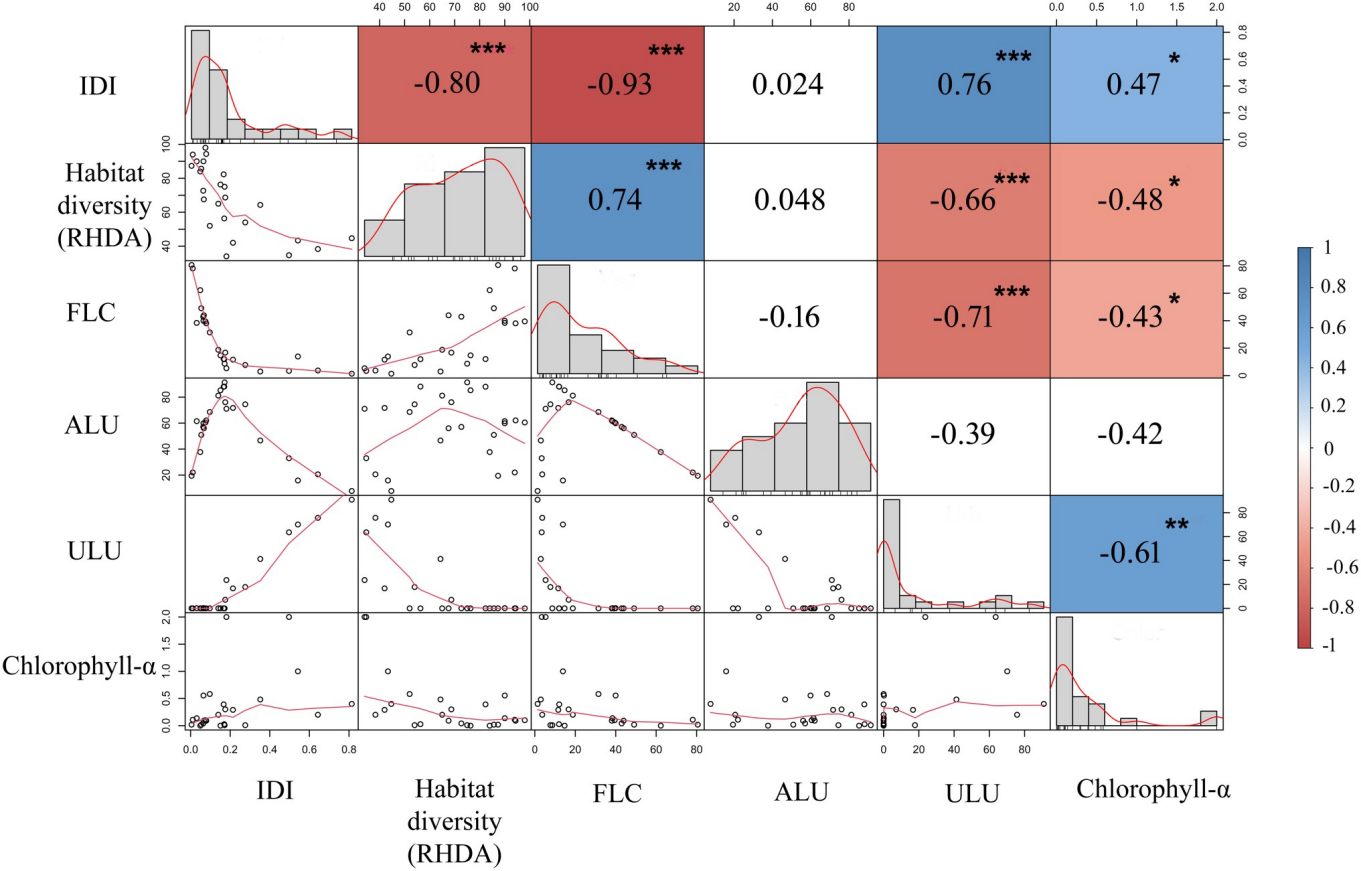
Spearman correlations among percentage of land use (%) (FLC—Forest Land Cover; ALU—agricultural land use; ULU—urban land use), Habitat diversity, Integrated Disturbance Index- IDI, and productivity (chlorophyll-α biomass) from the 25 streams sampled in the Iguaçu, Piquiri, and Ivaí River Basins, Brazil. The values in the squares represent the correlations, asterisks indicate significant correlations, blank squares indicate no significant correlations.

Streams with high disturbance were negatively correlated with habitat diversity and forested cover and were positively correlated with urban land use and Chl-α (Table 1; Fig 2). Habitat diversity was positively correlated with forested cover and negatively correlated with urban land use and Chl-α (Fig 2). Agricultural land use had no significant relationship with any of the evaluated variables.

**Table 1:** Characteristics of the sampled streams.

SC	Lat (S)	Long (W)	FLC	ALU	ULU	RHDA	IDI	Chl-α
**S1**	25°9’10.25"	53°16’41.86"	62.4	37.6	0.0	84.0	0.049	0.00
**S2**	25°6’7.17"	53°18’42.25"	76.0	24.0	0.0	94.0	0.011	0.11
**S3**	25°4’6.94"	53°13’59.64"	50.0	50.0	0.0	85.6	0.054	0.02
**S4**	25°0’43.32"	53°19’50.53"	25.2	71.3	3.5	68.6	0.175	0.30
**S5**	25°4’48.38"	53°24’2.86"	27.0	73.0	0.0	52.0	0.097	0.58
**S6**	25°7’1.29"	53°10’34.81"	15.9	84.1	0.0	56.3	0.170	0.03
**S7**	25°0’1.33"	53°28’45.86"	14.9	51.0	34.0	64.3	0.352	0.48
**S8**	24°59’8.69"	53°26’7.24"	16.5	0.8	82.7	43.3	0.542	1.29
**S9**	24°59’3.28"	53°28’30.18"	3.0	0.0	97.0	44.6	0.814	0.40
**S10**	23°53’10.28"	52°49’19.46"	80.3	19.7	0.0	87.3	0.005	0.02
**S11**	24°58’52.07"	53°16’15.76"	42.4	57.4	0.2	90.0	0.031	0.14
**S12**	25°4’9.57"	53°3’25.78"	46.1	52.9	0.0	72.6	0.064	0.04
**S13**	25°4’39.91"	53°5’11.88"	44.9	55.1	0.0	90.0	0.065	0.56
**S14**	24°34’15.67"	52°18’29.54"	44.8	55.2	0.0	86.6	0.067	0.09
**S15**	25°4’12.71"	53°11’22.60"	16.9	83.1	0.0	82.3	0.167	0.39
**S16**	23°55’31.76"	52°42’42.63"	20.7	79.3	0.0	76.3	0.151	0.00
**S17**	24°18’20.71"	52°31’31.75"	15.8	59.8	24.4	42.0	0.214	0.29
**S18**	24°55’47.43"	53°24’33.90"	4.5	10.1	85.5	38.3	0.643	0.20
**S19**	23°46’50.25"	53°17’34.90"	5.9	18.3	75.8	34.6	0.497	1.79
**S20**	24°27’59.15"	52°17’39.43"	53.4	46.6	0.0	98.0	0.075	0.10
**S21**	23°48’6.08"	52°44’18.39"	46.9	53.1	0.0	94.3	0.079	0.09
**S22**	24°23’51.76"	52°22’24.70"	20.1	79.9	0.0	65.0	0.140	0.20
**S23**	23°57’37.83"	52°37’57.20"	17.2	82.8	0.0	75.0	0.172	0.01
**S24**	23°45’54.66"	53°4’8.79"	9.0	60.7	30.4	34.0	0.181	2.43
**S25**	24°1’36.38"	52°22’47.29"	13.3	48.3	38.4	54.0	0.276	0.01

SC—stream code; geographic coordinates: Lat (S)–latitude, Long (W)–longitude; land cover and land use percentages: FLC–Forest Land Cover, ALU–Agricultural Land Use, ULU–Urban Land Use; RHDA (Rapid Habitat Diversity Assessment); IDI (Integrated disturbance index); and Chl-α (Chlorophyll-α, ug/L) values from the 25 streams sampled in the Iguaçu, Piquiri, and Ivaí River Basins, Brazil. Streams codes are according to S1 Table.

### Effects of the human pressure on species composition

A total of 13,615 individuals belonging to 63 species, 12 families, and six orders were sampled. Siluriformes were highlighted with greater species richness (29), followed by Characiformes (23 species). Characidae and Loricariidae were the families with the highest species richness (15 and 10, respectively). Eleven species were classified as non-native (S2 Table).

**Fig 3:**
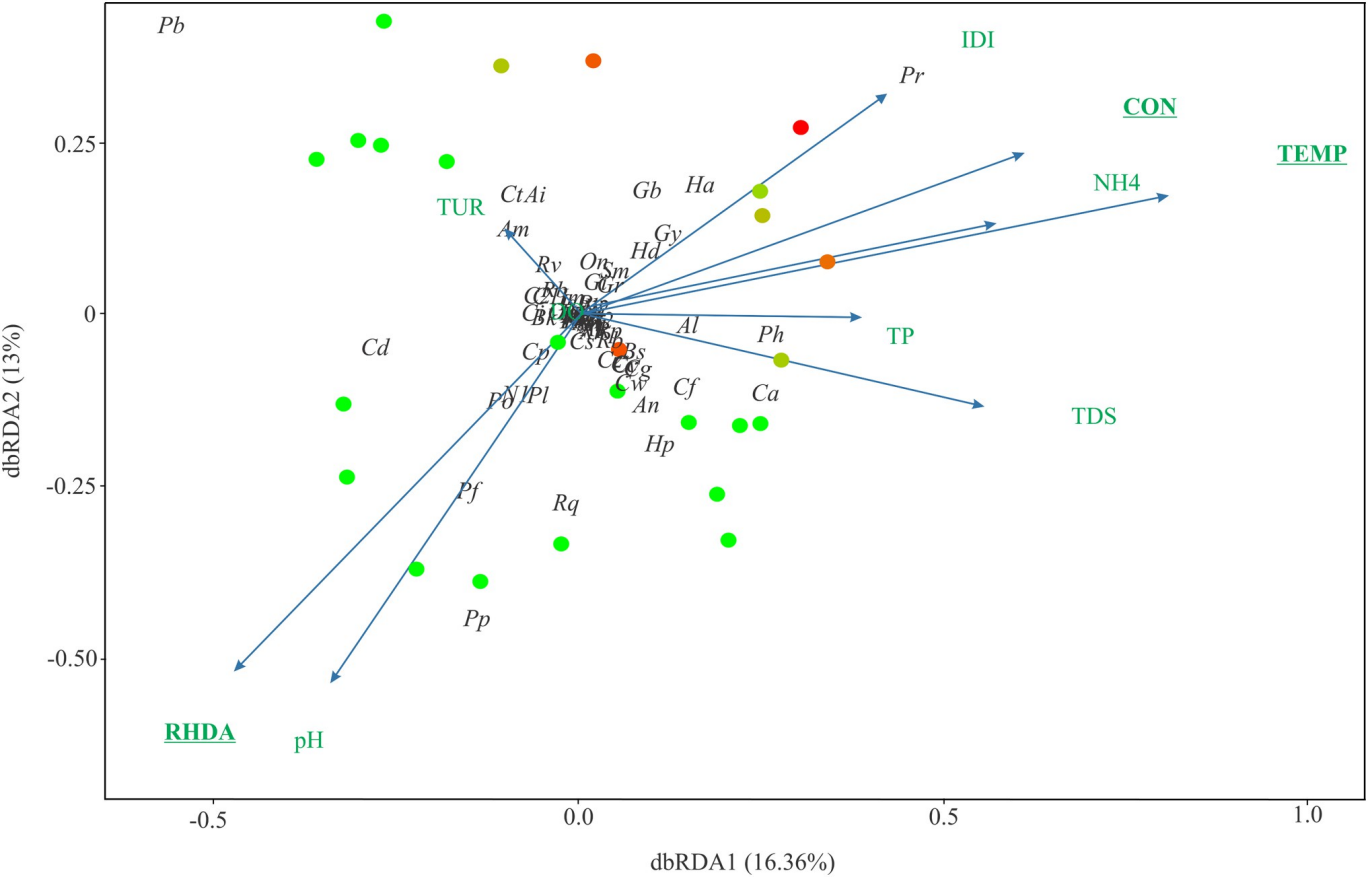
Distanced-based redundancy analyses (dbRDA) plots of species composition and the environmental variables for all streams. The colors demonstrate the degree of urbanization (red), that is, when the point is red, it presents a high degree of urbanization. The arrows indicate how the variables are related to the dbRDA axes and the underlined variables were statistically significant. IDI- Integrated Disturbance Index, RHDA- Rapid Habitat Diversity Assessment, TDS- Total dissolved solids, TP- Total phosphorus, NH4- Ammonia, TPT- Temperature, CON- Conductivity, DO- Dissolved oxygen, TUR- turbidity. Am = *Ancistrus mullerae*; An = *Ancistrus* sp.; Al = *Astyanax lacustris*, Cd = *Cambeva davisi*; Cf = *Cambeva* aff. *davisi*; Ct = *Cambeva stawiarski*; Ca = *Corydoras aeneus*; *Geophagus brasiliensis*; Gy = *Gymnotus sylvius* Hp = *Hisonotus pachysarkos*; Ha = *Hypostomus ancistroides*; Hd = *Hypostomus derbyi*; N1 = *Neoplecostomus* sp. 1; Pb = *Psalidodon bifasciatus*; Po = *Psalidodon bockmanni*; Pf = *Psalidodon* aff. *fasciatus*; Pp = *Psalidodon* aff. *paranae*; Ph = *Phalloceros harpagos*; Pr = *Poecilia reticulata*. See S2 Table for the code for the other species.

The first two axes of the dbRDA explained 29.36% of the variation, but significant differences were observed only for the dbRDA axis 1 (16,36%) (Axis 1 –F = 4.57; p = 0.048; Axis 2 –F = 3.28; p = 0.338). Temperature, conductivity, and RHDA explained the variability in the composition of fish fauna (Table 2). The first axis was positively correlated with temperature and conductivity, and negatively correlated with RHDA. Among the species that showed a positive correlation with the first axis of the dbRDA, 10 species are native for all sampling basins (*Ancistrus mullerae*, *Astyanax lacustris*, *Cambeva davisi*, *Cambeva* aff *davisi*, *Cambeva stawiarski*, *Corydoras aeneus*, *Geophagus brasiliensis*, *Hypostomus derbyi*, *Ancistrus* sp., *Hisonotus pachysarkos*), and two species are non-native to the Iguaçu River basin (*Hypostomus ancistroides*, *Gymnotus sylvius*) (Fig 3). Negative correlation was observed for six native species for all sampling basin (*Neoplecostomus* sp. 1, *Psalidodon* aff. *fasciatus*, *Psalidodon* aff. *paranae*, *Psalidodon bockmani*, *Psalidodon bifasciatus*, *Phalloceros harpago*) and one non-native species for all sampling basin (*Poecilia reticula*) (Fig 3).

**Table 2:** Relationships between species composition and explanatory variables in all streams based on distance-based redundancy analysis (dbRDA).

Explanatory variables	r² adjusted	F	p
pH	0.03	1.35	0.162
Temperature	0.08	1.96	0.033
Dissolved oxygen	-0.009	0.95	0.497
Conductivity	0.04	2.43	0.005
Total dissolved solids	0.03	1.47	0.143
Turbidity	-0.004	1.33	0.15
NH_4_ –Ammonia	0.04	1.27	0.219
Total phosphorus	0.009	0.81	0.636
Integrated Disturbance Index	0.03	1.05	0.362
Rapid Habitat Diversity Assessment	0.05	2.09	0.025

*p* values in bold highlight significant relations.

Indicator species analysis showed that, among the 63 species considered, only a few species were significantly related with disturbance levels. Four species were indicator species of streams with lower disturbance (*P*. *bifasciatus*, *A*. *minor*, *C*. *stawiarski* and *A*. *mullerae*), one of the streams with medium disturbance (*R*. *quelen*), and five were indicator species of streams with high disturbance, non-native, or resilient species (*P*. *reticulata*, *H*. *ancistroides*, *S*. *marmoratus*, *G*. *brasiliensis* and *H*. *derbyi*; Table 3). Considering the regional species pool, some species were emphasized as taxonomically rare by the restrictedness metric: *Apareiodon vladii*, *Psalidodon* aff. *gymnodontus*, *Bryconamericus ikaa*, *Callichthys callichthys*, *Cambeva mboycy*, and *Hoplias mbigua* (all of them with restrictedness = 0.96). Except for *Cambeva* cf. *mboycy*, these species occurred in streams with no urban influence.

**Table 3:** Species indicators defined by IndVal analysis, performed for each stream according to the IDI levels.

Stream’s category	Species	IndVal	*p*
Low disturbance	*P*. *bifasciatus*	0.98	0.001
	*A*. *minor*	0.84	0.029
	*C*. *stawiarski*	0.83	0.02
	*A*. *mullerae*	0.75	0.017
Medium disturbance	*R*. *quelen*	0.87	0.024
	*P*. *reticulata*	1.00	0.001
	*H*. *ancistroides*	0.80	0.032
High disturbance	*S*. *marmoratus*	0.70	0.013
	*G*. *brasiliensis*	0.66	0.015
	*H*. *derbyi*	0.65	0.024

IDI levels: < 0.09 values–low disturbance (sites with conditions closer to the natural), 0.10 to 0.19 –medium disturbance (altered sites), > 0.2 –high disturbance (extremely impacted sites).

**Fig 4:**
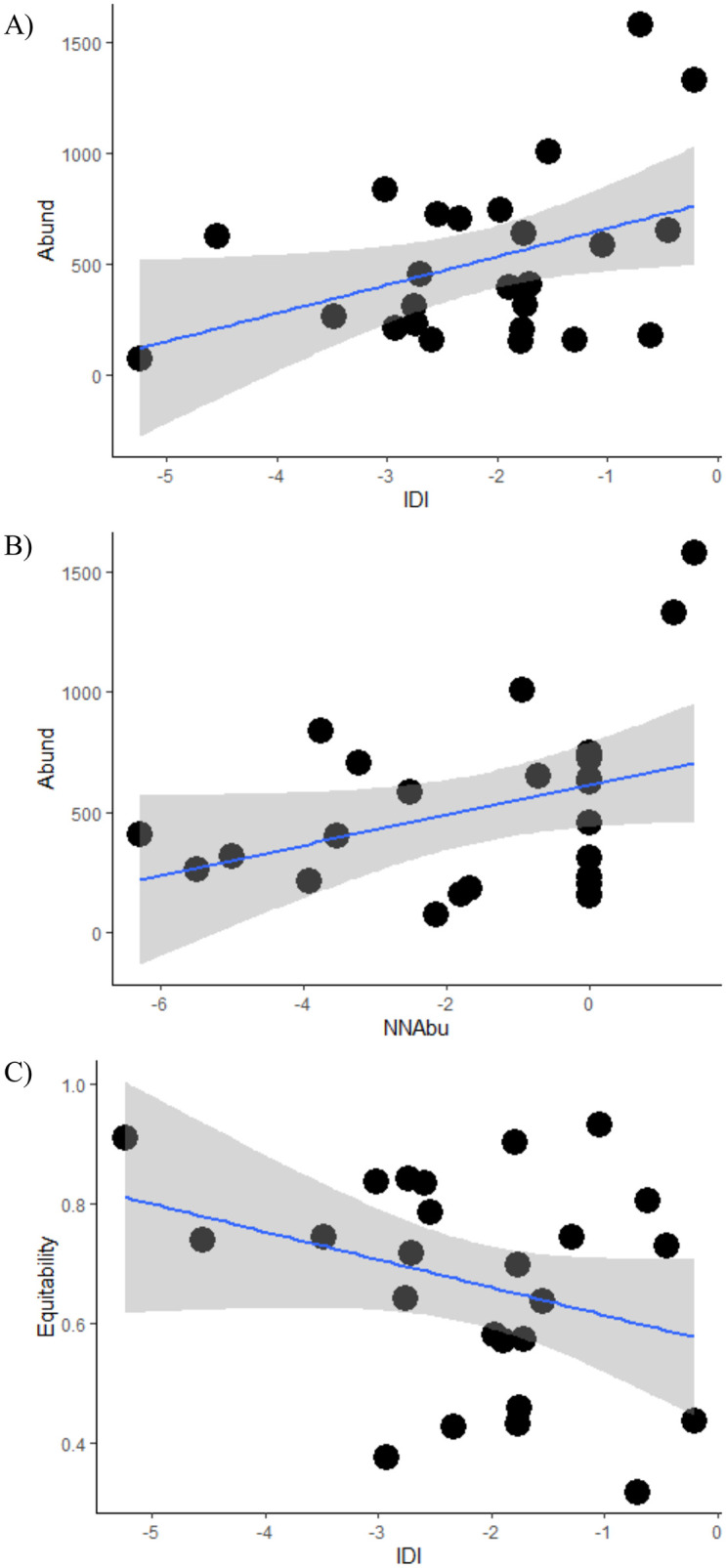
Relationships among the effect factors (x-axis) (log-transformed values) and metrics of fish assemblages’ structure (response variables, y-axis). The blue line represents the best fit of the Generalized Linear Mixed Models (GLMMs) to the data, and the gray shading area indicates the 95% confidence interval. **A.** Relations among numeric abundance (Abund) and Integrated Disturbance Index (IDI); **B.** Relations among numeric abundance (Abund) and non-native fish species abundance (NNAbu); **C.** Relations among equitability (Joule index) and IDI.

Previous influences of human pressure on fish species distribution are also observed in the fish fauna structure (Fig 4; Table 4). The numerical abundance of fish species was positively influenced by IDI (t = 2.662; p = 0.014) and NNabu (t = 2.396; p = 0.025) (Fig 4A and 4B). In general, streams with higher numerical abundance exhibited great disturbances and non-native fish abundance. Equitability was negatively affected by IDI (z = -1.933; p = 0.053) (Fig 4C; Table 4). However, species richness, species diversity, and Simpson’s dominance were not significantly affected by IDI and NNAbu.

**Table 4:** Effects of the Integrated Disturbance Index (IDI), and numeric abundance proportion of non-native fish species (NNAbu) on the metrics of fish assemblage’s structure evaluated in the Generalized Linear Mixed Models (GLMMs).

Response variable	Equitability	Abundance	
Effect factors	IDI	IDI	NNAbu
CI (2.5–97.5%)	-0.62–0.004	54.70–311.81	39.72–320.37
Estimated coefficient	-0.30888	183.26	180.05
Standard Error	0.15982	68.83	75.14
t value	-1.933*	2.662	2.396
p value	0.0533	0.0146	0.0259
Distribution (function used)	Beta (glmmTMB)*z value	Normal (lmer)	Normal (lmer)

Only significant relationships (p≤0.05) are presented. CI = Confidence intervals.

## Discussion

The results of this study demonstrated an inverse relationship between habitat diversity and disturbance. The intensification of human pressure on stream environments decreased habitat diversity and triggers changes in primary productivity, fish species composition, and assemblage structure. Streams with intense urban land use tended to present high productivity and low habitat diversity, which can culminate in habitat homogenization and eutrophication, drastically reducing the environmental quality in these environments. In disturbed streams, we observed the prevalence of the fish species previously reported in the literature as resistant to degradation; in contrast to the registered in the less disturbed streams, where the more sensitive, rare and endemic species prevailed. This relationship between resistant species and highly disturbed streams has been observed in other studies that evaluated human pressure on stream environments [72, 73]. In agreement with the initial hypothesis, the structure of the fish assemblage was affected by human pressure. Specifically, we verified that disturbance intensification tended to increase the numerical abundance and decreased the equitability in these fish assemblages. All these results corroborate that dominance represents a strong predictor of changes in communities induced by the main anthropogenic stressors, as highlighted by Hillebrand *et al*. [74].

### Effects of land use on stream habitats

Land use for human activities is intrinsically linked to ecological conditions in stream environments. Forest cover removal (on a catchment scale) and riparian forest (on a local scale) increases the input of sediments and nutrients in the stream channel, and intensifies erosion processes, mainly at the stream margins [12]. Such disturbances caused by human pressure were measured in the current study, presented via the IDI. The negative relationship among disturbances caused by land use (both on catchment and local scale) and habitat diversity in stream environments was clearly shown in the results. Therefore, regardless of the analyzed basins, reduction in habitat diversity can be used as a proxy for anthropogenic effects on stream ecosystems. The findings corroborate other studies showing the consequent homogenization of habitats induced by changes in land use from human activities [10, 16].

Primary productivity in streams is directly related to the environmental characteristics (physico-chemical conditions) of these ecosystems [75]. In our study, the correlations showed that in catchments with high urban land use, forested cover decreased, reducing habitat diversity and increasing primary productivity. In contrast, greater forested cover in streams catchment was positively related to greater habitat diversity. Thus, changes mediated by human pressure (high urban land use and low forested cover) in streams are evidenced by high disturbance (high IDI) and greater primary productivity. Variations in abiotic conditions, such as water temperature, pH, and nutrient load, significantly affect biological productivity in freshwater ecosystems [76]. In addition, substrate conditions can also influence chemical characteristics, especially in streams [76]. Primary productivity was generally low in all the sampled streams, but the few high values of productivity that were observed were only recorded in streams with high urban land use in their catchments. The decrease in shading due to the absence or reduction of riparian forest increase the exposure of the water surface to sunlight, and the water temperature increases [77]. High water temperature together with the nutrient’s enrichment provide conditions for an increase in primary productivity [75, 78], which can indicate a highly productive eutrophic state. The trophic state is fundamental to the ecosystem structure and is directly linked to the water quality and biotic integrity of streams [75]. The observed relationships among RHDA, temperature, conductivity, and dissimilarities in species composition corroborate the relationship between water quality and biotic integrity. Therefore, changes in primary productivity are predicted to mediate the food webs, also driving the composition and structure of the fish fauna.

### Individual species responses and identification of indicator species

Sets of distinct fauna that were strongly related to habitat diversity and disturbance gradients were verified in the analyzed streams. *Psalidodon bifasciatus* and *C*. *stawiarski* were mainly related to habitat diversity, corroborating their preference for resources that are more abundant in preserved streams. *Psalidodon* species feed mainly on plants, algae, and insects, and *Cambeva* species prefer reaches with riffles, consuming autochthonous food items, mainly insects [20, 79]. In contrast, degradation-resistant species, some of them non-native species, such as *G*. *sylvius* and *H*. *ancistroides* in the Iguaçu Basin [41], and *P*. *reticulata* [39, 53, 54], and even native species, such as *P*. *harpagos*, *C*. *aeneus*, and *A*. *lacustris*, were related to low habitat diversity. Such species can survive in environments with low oxygen levels and are trophic opportunistic, which provides resistance in altered environments [80–82].

Different species were highlighted as indicators in the sampled streams, considering the disturbance level. *Astyanax minor* and *A*. *mullerae*, endemic to the Iguaçu Basin, were indicative of forested stream with low disturbance. These species have requirements for habitat use and food [41] and have been reported to be sensitive to environmental degradation [9, 83]. In addition, *P*. *bifasciatus* and *C*. *stawiarski* were associated with low-disturbance streams. *Rhamdia quelen* was indicative of streams with medium disturbance; it is described as a species that lives in pools with sand and mud bottoms and is resistant to environmental variations such as pH and water temperature [84]. Agricultural land use in catchments generally modifies the substrate composition, reducing the presence of pebbles and gravel and increasing sedimentation. Such changes in the substrate lead to the predominance of soft bottoms in streams [85], which also alters species composition. The findings reported here corroborate those of other studies [86, 87], and reinforce the importance of assessing local and catchment conditions simultaneously with the historical conditions. Furthermore, it is noteworthy that these findings were independent of the analyzed basin and soil types, which indicates the great threat of the loss of ecosystem function to which these environments are exposed.

Degradation-resistant species were highlighted as species indicative of the highly disturbed streams group. *Poecilia reticulata* has been commonly associated with environments impacted by human activities, as registered in Brazilian streams altered by urbanization [3, 22, 88, 89]. *Hypostomus derbyi*, *H*. *ancistroides*, and *Synbranchus marmoratus* are resistant to low oxygen levels because they are considered stomach air-breathing [90, 91]. *Geophagus brasiliensis* is a generalist species that is tolerant to variations in temperature, pH, and low oxygen levels [92]. These biological traits allow these species to survive in disturbed environments, as indicated by the IndVal results that highlighted them as indicative of streams with high disturbance.

Native and endemic species were highlighted as rare species, by the taxon restrictedness metric, in the basins sampled. For example, *Apareiodon vladii* is an endemic species of the Piquiri and Ivaí river basins [54] and was sampled only in a forested stream during this study. *Apareiodon* species are trophic specialists that mainly feed on vegetal resources, with benthopelagic habit [9], and prefer habitats with high flow, well-oxygenated waters, and rocky substrates [93]. Registered only in an agricultural stream of the Ivaí Basin, *C*. *callichthys* is widespread in South American rivers [54], can breathe air to survive in hypoxic and shallow waters [94], and uses the aquatic vegetation accumulated on the riverbanks and swamps to lay its eggs [95], characteristics observed at the sampling site of their capture. *Hoplias mbigua*, *P*. aff. *gymnodontus*, *B*. *ikaa*, and *C*. *mboycy* were sampled from forested streams (except *C*. *mboycy*, urban stream) of the Iguaçu Basin and stand out in the regional species pool. Excluding *H*. *mbigua*, which is widely registered in freshwater environments in the Paraná–Paraguay system [54], the other species are considered endemic to the Iguaçu Basin [39]. Notably, *C*. *mboycy* inhabits the reaches with riffles and consume autochthonous food items, mainly insects, which are abundant resources in more preserved streams. In addition, this species was captured in low abundance in a stream that showed urbanization in the basin. This result reinforces the role of the riparian forest, which is fundamental to the physical structure, energy flow, and species diversity of this environment. Additionally, *C*. *mboycy* was categorized as endangered [96], indicating the fragility of this native species and the importance of preserving these streams. It is fundamental to expand the management and conservation efforts in this basin, mainly for the maintenance of riparian forests and habitat diversity, which is essential for preserving this fish fauna.

### Assemblage-level responses

High disturbance caused by human pressure in stream environments, such as land use intensification and non-native fish species introduction, positively affected the abundance of fish species. A high abundance of fish species has previously been related to impacted sites [9, 97], corroborating our results. Non-native fish species can drive species dominance and cause changes in the original composition of the fish species [98]. Such alterations could have occurred in the sampled urban streams, where there was a high abundance of *P*. *reticulata*. In the case of urbanization, the intensification of disturbances facilitates an increase in degradation-resistant species and a reduction in degradation-sensitive species, leading to the dominance of a few species [89, 99]. The existence of different stressors in the same stream or catchment drastically changes the composition and structure of the fish assemblage fish [79].

Equitability tended to decrease with disturbance intensification probably due the higher abundance of non-native fish species in the streams. Although the evenness index does not reflect whether the dominant species differs in important traits compared to the rare species [74], here we highlight the occurrence of non-native species resistant to degradation. These non-native species are generalist functional groups [72] that are degradation-resistant and proliferate rapidly dominating the fish assemblage. Thus, changes resulting from environmental conditions such as eutrophication favor resistant species with consequent changes in interactions and coexistence between species (competition for resources), resulting in reduced equitability. In streams under human pressure, the equitability responds rapidly to the occurred changes, which shows the importance to measure it when evaluating the effects of land use on fish assemblages [74]. It is worth mentioning that high disturbance was directly related to low habitat diversity and eutrophication (nutrient load + Chl-α), which are considered important factors in determining the structure of fish assemblages in freshwater ecosystems [100]. Greater disturbance reduces habitat availability for prey and food resources [101], which can cause an imbalance in species abundance, affecting the equitability and therefore, disruption of food webs in these assemblages, and even ecosystem processes [74]. Here, we emphasize that the synchronism between habitat degradation caused by land use and the introduction of non-native species enhances the deleterious effects on sensitive species, with consequent homogenization of the biota. In this respect, although the causes of species introduction are sometimes different from those that occur in large systems [9, 36], the effects on stream fish structure appear to be convergent.

Herein, this study stands out for comprising representative areas of two ecoregions. Our results suggest some perspectives underlying the current scenario of human impacts and the loss of Neotropical freshwater fish diversity. Considering that small-bodied fish, most of which are exclusive to streams, are considered the largest and most threatened portion of the megadiverse fauna of Neotropical freshwater fish [102], and that the disturbances reported here are predicted to increase (e.g., with the increase in urban areas), irreversible losses are inevitable. We can infer that regardless of the fish species richness of the basins (the Iguaçu River is comparatively poor in species richness), urbanization is a strong driver of productivity, species composition and structure, which can lead to fauna homogenization. Thus, land use and management decisions, as well as the culture of society, will be decisive in the conservation of stream biodiversity.

## Conclusion

In summary, the current study reinforces the important role of forest cover and habitat diversity in maintaining native, endemic, taxonomically rare, and degradation-sensitive fish assemblages in streams. Disturbance intensification drives the increase in primary productivity, as well as alterations in the composition and structure of fish fauna, leading to higher abundance and lower equitability, with the predominance of degradation-resistant species in the disturbed streams. The increase in non-native species abundance in the disturbed streams is also a driver of the higher abundance in the streams that were sampled. Headwater streams shelter a great number of endemic species, registered even in urban streams, a fact that runs contrary to conservation in these water bodies, where intensive disturbance can render endemic species extinct and make way for non-native species. Considering that the evaluated disturbances can lead to extirpation of sensitive species and that these species, mainly in the Iguaçu River Basin, are endemic and taxonomically restricted to this basin, such exclusion can mean their global extinction. Thus, it is necessary to enhance conservation efforts directed toward stream ecosystems to maintain or recover their biodiversity and ecosystem services.

## Supporting information

S1 Table Characteristics of the sampling areas (streams, surrounding of sampled streams and river basins). (DOCX)

S2 Table Fish species recorded in the streams from Iguaçu, Ivaí and Piquiri river basins, Brazil. (DOCX)

S1 File Protocol for Rapid Habitat Diversity Assessment (RHDA, applied in stretches of sampling sites during this study). (PDF)
