# Supplementary material for: Effects of human disturbance on habitat and fish diversity in Neotropical streams
Source: PLoS One. 2022 Sep 9;17(9):e0274191. doi: 10.1371/journal.pone.0274191 (PMC9462761; doi:10.1371/journal.pone.0274191)
Supplement: S1 Table — (DOCX) [file pone.0274191.s001.docx]

# Effects of human disturbance on habitat and fish diversity in Neotropical streams

Crislei Larentis¹^¶^, Bruna Caroline Kotz Kliemann²^¶^, Mayara Pereira Neves³^¶^ and Rosilene Luciana Delariva^4¶*^

¹Programa de Pós-Graduação em Biologia Comparada, Universidade Estadual de Maringá, Maringá, Paraná, Brazil.

²Programa de Pós-graduação em Ciências Biológicas/Zoologia, Instituto de Biociências, Universidade Estadual Paulista (UNESP), Botucatu, São Paulo, Brazil.

³Programa de Pós-graduação em Biologia Animal, Universidade Federal do Rio Grande do Sul, Porto Alegre, Rio Grande do Sul, Brazil.

^4^Laboratório de Ictiologia, Ecologia e Biomonitoramentos (LIEB), Universidade Estadual do Oeste do Paraná – UNIOESTE, Cascavel, Paraná, Brazil.

*Corresponding author

Email: [rosilene.delariva@unioeste.br](mailto:rosilene.delariva@unioeste.br,) (RD)

^¶^These authors contributed equally to this work.

**S1 Table. Characteristics of the sampling areas (streams, surrounding of sampled streams and river basins).**

| Code | Streams | Hyd. basin | Geographic coordinates | Basin  Área  (Km²) | Order (Strahler) | Land cover and land use  (%) | | | Land cover and land use surrounding of sampled streams | | | Characterization of river basins | | | | |
| --- | --- | --- | --- | --- | --- | --- | --- | --- | --- | --- | --- | --- | --- | --- | --- | --- |
|  |  |  |  |  |  | FLC | ALU | ULU | Forested area | Rural area | Urban area | Agricultural activities | Industrial development | Human population | Preservation areas |  |
| S1 | Arquimedes | Iguaçu | 25°9'10.25"S; 53°16'41.86"W | 6.77 | 3 | 62.4 | 37.6 | 0.0 | Sampling site in Forest Reserve | Sampling site in agricultural area - cereal crops | Sampling site - Cascavel municipality, with ≈ 324 mil inhabitants | Predomination of soybean, corn and wheat cultivation; livestock | Metropolitan region of Curitiba, and on region of Cascavel | Estimated in 3,9 million inhabitants | Approxima- tely 23 Conservation Areas, among which is the Iguaçu National Park. |  |
| S2 | Pedregulho | Iguaçu | 25° 6'7.17"S; 53°18'42.25"W | 6.27 | 2 | 76.0 | 24.0 | 0.0 |  |  |  |  |  |  |  |  |
| S3 | Rio do Salto | Iguaçu | 25° 4'6.94"S; 53°13'59.64"W | 13.62 | 2 | 50.0 | 50.0 | 0.0 |  |  |  |  |  |  |  |  |
| S4 | São José | Iguaçu | 25° 0'43.32"S; 53°19'50.53"W | 11.64 | 3 | 25.2 | 71.3 | 3.5 |  |  |  |  |  |  |  |  |
| S5 | Bom Retiro | Iguaçu | 25° 4'48.38"S; 53°24'2.86"W | 9.28 | 3 | 27.0 | 73.0 | 0.0 |  |  |  |  |  |  |  |  |
| S6 | Carolina | Iguaçu | 25° 7'1.29"S; 53°10'34.81"W | 7.32 | 2 | 15.9 | 84.1 | 0.0 |  |  |  |  |  |  |  |  |
| S7 | Afluente do Quati | Iguaçu | 25° 0'1.33"S; 53°28'45.86"W | 5.94 | 2 | 14.9 | 51.0 | 34.0 |  |  |  |  |  |  |  |  |
| S8 | Cascavel | Iguaçu | 24°58'35.77"S; 53°26'7.13"W | 12.63 | 3 | 16.5 | 0.8 | 82.7 |  |  |  |  |  |  |  |  |
| S9 | Quati | Iguaçu | 24°59'3.28"S; 53°28'30.18"W | 9.14 | 2 | 3.0 | 0.0 | 97.0 |  |  |  |  |  |  |  |  |
| S10 | Mouro | Piquiri | 23°53'10.28"S; 52°49'19.46"W | 48.52 | 4 | 80.3 | 19.7 | 0.0 | Ambiental Park of Cascavel, forest remnants and Rebio das Perobas Biological Reserve | Agricultural areas - cereal crops (soybean, corn) and livestock | Cascavel municipality, Mamborê mun. ≈13 mil inhabitants, and Umuarama mun. ≈110 mil inh. | Cultivation of soybeans, wheat, sugarcane and cassava; livestock | Related to farming, mainly dairy industry, and slaughter refrigerator | Approximately 620 mil inhabitants | Approxima- tely 80 Conservation Areas, among which is the Perobas Biological Reserve |  |
| S11 | Carreira | Piquiri | 24°58'52.07"S; 53°16'15.76"W | 18.25 | 2 | 42.4 | 57.4 | 0.2 |  |  |  |  |  |  |  |  |
| S12 | Barreiro | Piquiri | 25° 3'22.73"S; 53° 3'58.82"W | 16.24 | 3 | 46.1 | 52.9 | 0.0 |  |  |  |  |  |  |  |  |
| S13 | Ano Novo | Piquiri | 25° 4'39.91"S; 53° 5'11.88"W | 12.9 | 4 | 44.9 | 55.1 | 0.0 |  |  |  |  |  |  |  |  |
| S14 | Barro Preto | Piquiri | 24°34'15.67"S; 52°18'29.54"W | 9.14 | 2 | 44.8 | 55.2 | 0.0 |  |  |  |  |  |  |  |  |
| S15 | Piquirizinho | Piquiri | 25° 4'12.71"S; 53°11'22.60"W | 6.63 | 2 | 16.9 | 83.1 | 0.0 |  |  |  |  |  |  |  |  |
| S16 | Corrégo 52 | Piquiri | 23°55'31.76"S; 52°42'42.63"W | 23.39 | 4 | 20.7 | 79.3 | 0.0 |  |  |  |  |  |  |  |  |
| S17 | Sununu | Piquiri | 24°18'20.71"S; 52°31'31.75"W | 8.58 | 3 | 15.8 | 59.8 | 24.4 |  |  |  |  |  |  |  |  |
| S18 | Aroeira | Piquiri | 24°55'47.43"S; 53°24'33.90"W | 11.36 | 3 | 4.5 | 10.1 | 85.5 |  |  |  |  |  |  |  |  |
| S19 | Pinhalzinho II | Piquiri | 23°46'50.25"S; 53°17'34.90"W | 18.04 | 3 | 5.9 | 18.3 | 75.8 |  |  |  |  |  |  |  |  |
| S20 | Formosinho | Ivaí | 24°27'59.15"S; 52°17'39.43"W | 25.29 | 2 | 53.4 | 46.6 | 0.0 | Forest remnants, well-structured riparian forest, Rebio das Perobas B.R. | Crop-livestock integration, cereal crops, mainly soybean | Campo Mourão mun. ≈94 mil inh., and Cruzeiro do Oeste mun. ≈20 mil inh. | Cultivation of soybeans, corn, wheat, sugarcane; livestock | Related with agriculture and livestock | Approximately 1,3 million inhabitants | Approxima- tely 74 Conservation Areas; among which is the Perobas Biological Reserve |  |
| S21 | Ariranha | Ivaí | 23°48'6.08"S; 52°44'18.39"W | 5.99 | 2 | 46.9 | 53.1 | 0.0 |  |  |  |  |  |  |  |  |
| S22 | Sem Passo | Ivaí | 24°23'51.76"S; 52°22'24.70"W | 7.87 | 3 | 20.1 | 79.9 | 0.0 |  |  |  |  |  |  |  |  |
| S23 | Ligeiro | Ivaí | 23°57'37.83"S; 52°37'57.20"W | 10.25 | 3 | 17.2 | 82.8 | 0.0 |  |  |  |  |  |  |  |  |
| S24 | Rio das Antas | Ivaí | 23°45'54.66"S; 53° 4'8.79"W | 11.78 | 3 | 9.0 | 60.7 | 30.4 |  |  |  |  |  |  |  |  |
| S25 | Água Km119 | Ivaí | 24° 1'36.38"S; 52°22'47.29"W | 28.68 | 3 | 13.3 | 48.3 | 38.4 |  |  |  |  |  |  |  |  |

FLC= Forest Land Cover; ALU= Agricultural Land Use; ULU= Urban Land Use.
