# Supplementary material for: Effects of human disturbance on habitat and fish diversity in Neotropical streams
Source: PLoS One. 2022 Sep 9;17(9):e0274191. doi: 10.1371/journal.pone.0274191 (PMC9462761; doi:10.1371/journal.pone.0274191)
Supplement: S2 Table — (DOCX) [file pone.0274191.s002.docx]

# Effects of human disturbance on habitat and fish diversity in Neotropical streams

Crislei Larentis¹^¶^, Bruna Caroline Kotz Kliemann²^¶^, Mayara Pereira Neves³^¶^ and Rosilene Luciana Delariva^4¶*^

¹Programa de Pós-Graduação em Biologia Comparada, Universidade Estadual de Maringá, Maringá, Paraná, Brazil.

²Programa de Pós-graduação em Ciências Biológicas/Zoologia, Instituto de Biociências, Universidade Estadual Paulista (UNESP), Botucatu, São Paulo, Brazil.

³Programa de Pós-graduação em Biologia Animal, Universidade Federal do Rio Grande do Sul, Porto Alegre, Rio Grande do Sul, Brazil.

^4^Laboratório de Ictiologia, Ecologia e Biomonitoramentos (LIEB), Universidade Estadual do Oeste do Paraná – UNIOESTE, Cascavel, Paraná, Brazil.

*Corresponding author

Email: [rosilene.delariva@unioeste.br](mailto:rosilene.delariva@unioeste.br,) (RD)

^¶^These authors contributed equally to this work.

**S2 Table.** **Fish species recorded in the streams from Iguaçu, Ivaí and Piquiri river basins, Brazil.**

**Protocolo de Avaliação Rápida da Diversidade de Habitats**

| **Taxon** | **Species code** | **Common name** | **Origin*** | **Voucher number** |
| --- | --- | --- | --- | --- |
| **OSTEICHTHYES** |  |  |  |  |
| **CHARACIFORMES** |  |  |  |  |
| **Characidae** |  |  |  |  |
| *Bryconamericus ikaa* Casciotta, Almirón & Azpelicueta, 2004 | Bk | “Lambari” | Native | UFRGS 26,246 |
| *Bryconamericus* sp. | Bs | "Lambari" | Native | NUP 21206, 21210, 21216 |
| *Hyphessobrycon* sp. | Hb | "Mato-Grosso" | Nonnative Piquiri Basin | NUP 21229 |
| *Serrapinnus notomelas* (Eigenmann, 1915) | Sn | "Lambari, Piabinha" | Native | NUP 21182, 21208, 21233 |
| ***Insertae sedis*** |  |  |  |  |
| *Astyanax* sp. 1 | A1 | “Lambari” | Native | NUP 21227 |
| *Astyanax* sp. 2 | A2 | “Lambari” | Native |  |
| *Psalidodon* aff. *gymnodontus* (Eigenmann, 1911) | Pg | “Lambari” | Native | UFRGS 25,725 |
| *Psalidodon* aff. *fasciatus* (Cuvier, 1819) | Pf | "Lambari-do-rabo-vermelho" | Native | NUP 21207, 21209, 21212, 21217, NUP 21231 |
| *Psalidodon* aff. *leonidas* Azpelicueta, Casciotta & Almirón, 2002 | Pl | "Lambari" | Native | NUP 21227 |
| *Astyanax lacustris* (Lütken, 1875) | Al | "Lambari-do-rabo-amarelo" | Native | NUP 21191, 21197 |
| *Astyanax minor* Garavello & Sampaio, 2010 | Ai | "Lambari" | Native | NUP 19026 |
| *Psalidodon* aff. *paranae* (Eigenmann, 1914) | Pp | "Lambari" | Native | NUP 21204, 21205, 21232, UFRGS 26,232 |
| *Psalidodon bifasciatus* (Garavello & Sampaio, 2010) | Pb | "Lambari-do-rabo-vermelho" | Native | NUP 21222, UFRGS 26,235 |
| *Psalidodon bockmanni* Vari, Castro, 2007 | Po | "Lambari" | Native | NUP 21228 |
| *Oligosarcus paranensis* Menezes, Géry, 1983 | Op | "Peixe-cachorro" | Native | NUP 21156 |
| **Crenuchidae** |  |  |  |  |
| *Characidium* sp. | Cp | "canivete, mocinha" | Native | NUP 21185 |
| *Characidium* aff. *zebra* Eigenmann, 1909 | Cz | "canivete, mocinha" | Native | NUP 21186, 21189 |
| *Characidium gomesi* Travassos, 1956 | Cg | "canivete, mocinha" | Native | NUP 21187, 21188 |
| **Parodontidae** |  |  |  |  |
| *Apareiodon vladii* Pavanelli, 2006 | Av | "canivete" | Native | NUP 21162 |
| **Erythrinidae** |  |  |  |  |
| *Hoplias* sp. 1 | H1 | "Traíra" | Native | NUP 21802 |
| *Hoplias* sp. 2 | H2 | "Traíra" | Native | NUP 21234 |
| *Hoplias* *argentinensis* Rosso, González-Castro, Bogan, Cardoso, Mabragaña, Delpiani, Días de Astarloa, 2018 | Hr | "Traíra" | Native | NUP 21166, 21167 |
| *Hoplias mbigua* Azpelicueta, Benítez, Aichino, Mendez, 2005 | Hm | "Traíra" | Native | NUP 21803 |
| **CICHLIFORMES** |  |  |  |  |
| **Cichlidae** |  |  |  |  |
| *Cichlasoma paranaense* Kullander, 1983 | Ch | "Acará paranaense" | Native | NUP 21155 |
| *Geophagus brasiliensis* (Quoy, Gaimard, 1824) | Gb | "Acará, cará" | Native | NUP 21157 |
| *Oreochromis niloticus* (Linnaeus, 1758) | On | "Tilápia-doNilo" | Nonnative Piquiri Basin | NUP 21153, 21154 |
| **CYPRINODONTIFORMES** |  |  |  |  |
| **Poeciliidae** |  |  |  |  |
| *Phalloceros harpagos* Lucinda, 2008 | Ph | "Guppies, Barrigudinho" | Native | NUP 21219 |
| *Poecilia reticulata* Peters, 1859 | Pr | "Guppies, Barrigudinho" | Nonnative Piquiri, Ivai and Iguaçu Basins | NUP 21218 |
| *Xiphophorus helleri* Heckel 1848 | Xh | “Espadinha” | Nonnative Iguaçu Basin | NUP 21119 |
| **GYMNOTIFORMES** |  |  |  |  |
| **Gymnotidae** |  |  |  |  |
| *Gymnotus inaequilabiatus* (Valenciennes, 1839) | Gi | "morenita, tuvira" | Nonnative Iguaçu Basin | NUP 21214 |
| *Gymnotus pantanal* Fernandes, Albert, Daniel-Silva, Lopes, Crampton, Almeida-Toledo, 2005 | Gp | "morenita, tuvira" | Nonnative Piquiri and Ivai Basin | NUP 21198, 21200, 21201, 21202 |
| *Gymnotus paraguensis* Albert, Crampton, 2003 | Gr | "morenita, tuvira" | Nonnative Piquiri, Ivai and Iguaçu Basins | NUP 21804, 21805 |
| *Gymnotus sylvius* Albert, Fernandes-Matioli, 1999 | Gy | "morenita, tuvira" | Nonnative Iguaçu Basin | NUP 21199 |
| **SILURIFORMES** |  |  |  |  |
| **Callichthyidae** |  |  |  |  |
| *Callichthys callichthys* (Linnaeus, 1758) | Cc | "Tambuatá, Tamoatá" | Native | NUP 21159, 21160, |
| *Corydoras aeneus* (Gill, 1858) | Ca | "Coridora" | Native | NUP 21181 |
| *Corydoras* cf. *lacrimostigmata* Tencatt, Britto & Pavanelli, 2014 | Cy | "Coridora" | Native | NUP 21196 |
| **Heptapteridae** |  |  |  |  |
| *Heptapterus* sp. | Hs | “Bagre das rochas” | Nonnative Iguaçu Basin | NUP 19036 |
| *Cetopsorhamdia iheringi* Schubart, Gomes, 1959 | Ci | "Bagrinho" | Native | NUP 21184 |
| *Imparfinis borodini* Mees, Cala, 1989 | Ib | "Bagrinho" | Native | NUP 21173, 21174 |
| *Imparfinis mirini* Haseman, 1911 | Im | "Bagrinho" | Native | NUP 21178 |
| *Imparfinis schubarti* (Gomes, 1956) | Is | "Bagrinho" | Native | NUP 21175, 21176, 21177 |
| *Rhamdia branneri* Haseman 1911 | Rb | "Bagre, Jundiá" | Native | NUP 19044 |
| *Rhamdia quelen* (Quoy, Gaimard, 1824) | Rq | "Bagre, Jundiá" | Native | NUP 21180, 21183 |
| *Rhamdia voulezi* Haseman, 1911 | Rv | "Bagre, Jundiá" | Native | NUP 21213 |
| **Loricariidae** |  |  |  |  |
| *Ancistrus* sp. | Na | "cascudinho" | Native | NUP 21161, 21230 |
| *Ancistrus mullerae* Bifi, Pavanelli & Zawadzki 2009 | Am | "cascudinho" | Native | NUP 19022 |
| *Hisonotus pachysarkos* Zawadzki, Roxo & Graça, 2016 | Hp | "cascudinho" | Native | NUP 21171 |
| *Hypostomus* sp. | Hy | "cascudo" | Native |  |
| *Hypostomus ancistroides* (Ihering, 1911) | Há | "cascudo" | Nonnative Iguaçu Basin | NUP 21172 |
| *Hypostomus derbyi* Haseman, 1911 | Hd | "cascudo" | Native | NUP 21215 |
| *Hypostomus nigromaculatus* (Schubart, 1964) | Hn | "cascudo" | Native | NUP 21168 |
| *Neoplecostomus* sp. 1 | N1 | "cascudinho" | Native | NUP 21163 |
| *Neoplecostomus* sp. 2 | N2 | "cascudinho" | Native | NUP 21164 |
| *Rineloricaria pentamaculata* Langeani & de Araujo, 1994 | Rp | "cascudo-chinelo" | Native | NUP 21169, 21170 |
| **Trichomycteridae** |  |  |  |  |
| *Cambeva* sp. | Cs | "Candiru" | Native | NUP 21190 |
| *Cambeva* sp. 1 | C1 | "Candiru" | Native | NUP 21223 |
| *Cambeva* sp. 2 | C2 | "Candiru" | Native | NUP 21225 |
| *Cambeva mboycy* (Wosiacki & Garavello 2004) | Cm | "Candiru" | Native | NUP 19051 |
| *Cambeva davisi* (Haseman, 1911) | Cd | "Candiru" | Native | NUP 21221, 21224 |
| *Cambeva* aff. *davisi* (Haseman, 1911) | Cf | "Candiru" | Native | NUP 21194, 21195 |
| *Cambeva stawiarski* (Miranda Ribeiro, 1968) | Cs | "Candiru" | Native | NUP 21220 |
| *Cambeva* aff. *stawiarski* (Miranda Ribeiro, 1968) | Cw | "Candiru" | Native | NUP 21192 |
| **SYNBRANCHIFORMES** |  |  |  |  |
| **Synbranchidae** |  |  |  |  |
| *Synbranchus marmoratus* Bloch, 1795 | Sm | "Mussum" | Nonnative Iguaçu Basin | NUP 21158 |

Specimens of all the sampled species were deposited in the Coleção Ictiológica do Nupélia (Núcleo de Pesquisas em Limnologia, Ictiologia e Aquicultura), on the Universidade Estadual de Maringá (Universidade Estadual de Maringá - UEM), Paraná State (NUP vouchers), and in the Coleção Ictiológica da Universidade Federal do Rio Grande do Sul (UFRGS vouchers). *Origin was categorized according to Baumgartner *et al*. (2012), Graça, Pavanelli (2007), and Ota *et al*. (2018).
