# Supplementary material for: Effects of human disturbance on habitat and fish diversity in Neotropical streams
Source: PLoS One. 2022 Sep 9;17(9):e0274191. doi: 10.1371/journal.pone.0274191 (PMC9462761; doi:10.1371/journal.pone.0274191)
Supplement: S1 File — (PDF) [file pone.0274191.s003.pdf]

Effects of human disturbance on habitat and fish diversity in Neotropical streams

Crislei Larentis[1¶], Bruna Caroline Kotz Kliemann[2¶], Mayara Pereira Neves[3¶] and Rosilene Luciana Delariva[4¶*]

[1]Programa de Pós-Graduação em Biologia Comparada, Universidade Estadual de Maringá, Maringá, Paraná, Brazil.
[2]Programa de Pós-graduação em Ciências Biológicas/Zoologia, Instituto de Biociências, Universidade Estadual Paulista (UNESP), Botucatu, São Paulo, Brazil.
[3]Programa de Pós-graduação em Biologia Animal, Universidade Federal do Rio Grande do Sul, Porto Alegre, Rio Grande do Sul, Brazil.
[4]Laboratório de Ictiologia, Ecologia e Biomonitoramentos (LIEB), Universidade Estadual do Oeste do Paraná – UNIOESTE, Cascavel, Paraná, Brazil.

*Corresponding author
Email: rosilene.delariva@unioeste.br

¶These authors contributed equally to this work.

S1 File. Protocol for Rapid Habitat Diversity Assessment (RHDA, applied in stretches of sampling sites during this study.

**DESCRIÇÃO DO AMBIENTE**
Localização:
Data da Coleta: ____/_____/_____ Hora da Coleta:
Tempo (situação do dia):
Modo de coleta (coletor):
Tipo de ambiente: Córrego ( ) Rio ( )
Largura média:
Profundidade média:
Temperatura da água:

PARÂMETROS	PONTUAÇÃO		
	**4 pontos**	**2 pontos**	**0 ponto**
**1.Tipo de ocupação das margens do corpo d'água (principal atividade)**	Vegetação natural	Campo de pastagem/Agricultura/ Monocultura/ Reflorestamento	Residencial/ Comercial/ Industrial
**2. Erosão próxima e/ou nas margens do rio e assoreamento em seu leito**	Ausente	Moderada	Acentuada
**3. Alterações antrópicas**	Ausente	Alterações de origem doméstica (esgoto, lixo)	Alterações de origem industrial/ urbana (fábricas, siderurgias, canalização, retilinização do curso do rio)
**4. Cobertura vegetal no leito**	Parcial	Total	Ausente
**5. Odor da água**	Nenhum	Esgoto (ovo podre)	Óleo/industrial
**6. Oleosidade da água**	Ausente	Moderada	Abundante
**7. Transparência da água**	Transparente	Turva/cor de chá-forte	Opaca ou colorida
**8. Odor do sedimento (fundo)**	Nenhum	Esgoto (ovo podre)	Óleo/industrial
**9. Oleosidade do fundo**	Ausente	Moderado	Abundante
**10. Tipo de fundo**	Pedras/cascalho	Lama/areia	Cimento/canalizado

PARÂMETROS	PONTUAÇÃO			
	**5 pontos**	**3 pontos**	**2 pontos**	**0 ponto**
**11. Tipos de fundo**	Mais de 50% com habitats diversificados; pedaços de troncos submersos;	30 a 50% de habitats diversificados; habitats adequados para a	10 a 30% de habitats diversificados;	Menos que 10% de habitats diversificados; ausência de habitats óbvia; substrato rochoso instável para

	cascalho ou outros habitats estáveis.	manutenção das populações de organismos aquáticos.	disponibilidade de habitats insuficiente; substratos frequentemente modificados.	fixação dos organismos.
**12. Extensão de Rápidos**	Rápidos e corredeiras bem desenvolvidas; rápidos tão largos quanto o rio e com o comprimento igual ao dobro da largura do rio.	Rápidos com a largura igual à do rio, mas com comprimento menor que o dobro da largura do rio.	Trechos rápidos podem estar ausentes; rápidos não tão largos quanto o rio e seu comprimento menor que o dobro da largura do rio.	Rápidos ou corredeiras inexistentes.
**13. Frequência de Rápidos**	Rápidos relativamente frequentes; distância entre rápidos dividida pela largura do rio entre 5 e 7.	Rápidos não frequentes; distância entre rápidos dividida pela largura do rio entre 7 e 15.	Rápidos ou corredeiras ocasionais; habitats formados pelos contornos do fundo; distância entre rápidos dividida pela largura do rio entre 15 e 25.	Geralmente com lâmina d’água “lisa” ou com rápidos rasos; pobreza de habitats; distância entre rápidos dividida pela largura do rio maior que 25.
**14. Tipos de Substrato**	Seixos abundantes (prevalecendo em nascentes).	Seixos abundantes; cascalho comum.	Fundo formado predominantemente por cascalho; alguns seixos presentes.	Fundo pedregoso; seixos ou lamoso.

**15. Deposição de Lama**	Entre 0 e 25% do fundo coberto por lama.	Entre 25 e 50% do fundo coberto por lama.	Entre 50 e 75% do fundo coberto por lama.	Mais de 75% do fundo coberto por lama.
**16. Depósitos Sedimentares**	Menos de 5% do fundo com deposição de lama; ausência de deposição nos remansos.	Alguma evidência de modificação no fundo, principalmente como aumento de cascalho, areia ou lama; 5 a 30% do fundo afetado; suave deposição nos remansos.	Deposição moderada de cascalho novo, areia ou lama nas margens; entre 30 a 50% do fundo afetado; deposição moderada nos remansos.	Grandes depósitos de lama, maior desenvolvimento das margens; mais de 50% do fundo modificado; remansos ausentes devido à significativa deposição de sedimentos.
**17. Alterações no canal do rio**	Canalização (retificação) ou dragagem ausente ou mínima; rio com padrão normal.	Alguma canalização presente, normalmente próximo à construção de pontes; evidência de modificações há mais de 20 anos.	Alguma modificação presente nas duas margens; 40 a 80% do rio modificado.	Margens modificadas; acima de 80% do rio modificado.
**18 Características do fluxo das águas**	Fluxo relativamente igual em toda a largura do rio; mínima quantidade de substrato exposta.	Lâmina d’água acima de 75% do canal do rio; ou menos de 25% do substrato exposto.	Lâmina d’água entre 25 e 75% do canal do rio, e/ou maior parte do substrato nos “rápidos” exposto.	Lâmina d’água escassa e presente apenas nos remansos.

**19. Presença de mata ciliar**	Acima de 90% com vegetação ripária nativa, incluindo árvores, arbustos ou macrófitas; mínima evidência de desflorestamento; todas as plantas atingindo a altura “normal”.	Entre 70 e 90% com vegetação ripária nativa; desfloresta-mento evidente mas não afetando o desenvolvimento da vegetação; maioria das plantas atingindo a altura “normal”.	Entre 50 e 70% com vegetação ripária nativa; desfloresta-mento óbvio; trechos com solo exposto ou vegetação eliminada; menos da metade das plantas atingindo a altura “normal”.	Menos de 50% da mata ciliar nativa; desfloresta-mento muito acentuado.
**20 Estabilidade das Margens**	Margens estáveis; evidência de erosão mínima ou ausente; pequeno potencial para problemas futuros. Menos de 5% da margem afetada.	Moderadamente estáveis; pequenas áreas de erosão frequentes. Entre 5 e 30% da margem com erosão.	Moderadamente instável; entre 30 e 60% da margem com erosão. Risco elevado de erosão durante enchentes.	Instável; muitas áreas com erosão; frequentes áreas descobertas nas curvas do rio; erosão óbvia entre 60 e 100% da margem.
**21. Extensão de mata ciliar**	Largura da vegetação ripária maior que 18 m; sem influência de atividades antrópicas (agropecuária, estradas, etc.).	Largura da vegetação ripária entre 12 e 18 m; mínima influência antrópica.	Largura da vegetação ripária entre 6 e 12 m; influência antrópica intensa.	Largura da vegetação ripária menor que 6 m; vegetação restrita ou ausente devido à atividade antrópica.
**22. Presença de plantas Aquáticas**	Pequenas macrófitas aquáticas e/ou musgos	Macrófitas aquáticas ou algas filamentosas ou musgos distribuídas no rio,	Algas filamentosas ou macrófitas em poucas pedras ou alguns remansos,	Ausência de vegetação aquática no leito do rio ou grandes bancos macrófitas

	distribuídos pelo leito.	substrato com perifiton.	perifiton abundante e biofilme.	(p.ex. aguapé).

Source: Callisto et al. (2002) - the first 10 parameters were adapted from the proposal of Ohio Environmental Protection Agency - USA (EPA,1987), and the other 12 parameters were adapted from the protocol presented by Hannaford et al. (1997).
